# Developing liver-targeted naringenin nanoparticles for breast cancer endocrine therapy by promoting estrogen metabolism

**DOI:** 10.1186/s12951-024-02356-0

**Published:** 2024-03-19

**Authors:** Yuying Zhao, Hanxu Tan, Juping Zhang, Dandan Zhan, Bowen Yang, Shicui Hong, Bo Pan, Neng Wang, Tongkai Chen, Yafei Shi, Zhiyu Wang

**Affiliations:** 1 State Key Laboratory of Dampness, Syndrome of Chinese Medicine, The Second Affiliated Hospital of Guangzhou University of Chinese Medicine, Guangzhou University of Chinese Medicine, Guangzhou, Guangdong China; 2 The Research Center for Integrative Medicine, School of Basic Medical Sciences, Guangzhou University of Chinese Medicine, Guangzhou, Guangdong China; 3 Guangdong-Hong Kong-Macau Joint Lab on Chinese Medicine and Immune Disease Research, Guangzhou University of Chinese Medicine, Guangzhou, Guangdong China; 4 Science and Technology Innovation Center, Guangzhou University of Chinese Medicine, Guangzhou, Guangdong China; 5 Guangdong Provincial Key Laboratory of Clinical Research On Traditional Chinese Medicine Syndrome, Guangdong Provincial Hospital of Chinese Medicine, Guangdong Provincial Academy of Chinese Medical Sciences, Guangzhou, Guangdong China

**Keywords:** Breast cancer, Endocrine therapy, Estrogen sulfotransferase, Naringenin, Nanodrug, Liver targeting

## Abstract

Endocrine therapy is standard for hormone receptor–positive (HR^+^) breast cancer treatment. However, current strategies targeting estrogen signaling pay little attention to estradiol metabolism in the liver and is usually challenged by treatment failure. In a previous study, we demonstrated that the natural compound naringenin (NAR) inhibited HR^+^ breast cancer growth by activating estrogen sulfotransferase (EST) expression in the liver. Nevertheless, the poor water solubility, low bio-barrier permeability, and non-specific distribution limited its clinical application, particularly for oral administration. Here, a novel nano endocrine drug NAR-cell penetrating peptide-galactose nanoparticles (NCG) is reported. We demonstrated that NCG presented specific liver targeting and increased intestinal barrier permeability in both cell and zebrafish xenotransplantation models. Furthermore, NCG showed liver targeting and enterohepatic circulation in mouse breast cancer xenografts following oral administration. Notably, the cancer inhibition efficacy of NCG was superior to that of both NAR and the positive control tamoxifen, and was accompanied by increased hepatic EST expression and reduced estradiol levels in the liver, blood, and tumor tissue. Moreover, few side effects were observed after NCG treatment. Our findings reveal NCG as a promising candidate for endocrine therapy and highlight hepatic EST targeting as a novel therapeutic strategy for HR^+^ breast cancer.

**Graphical Abstract:**

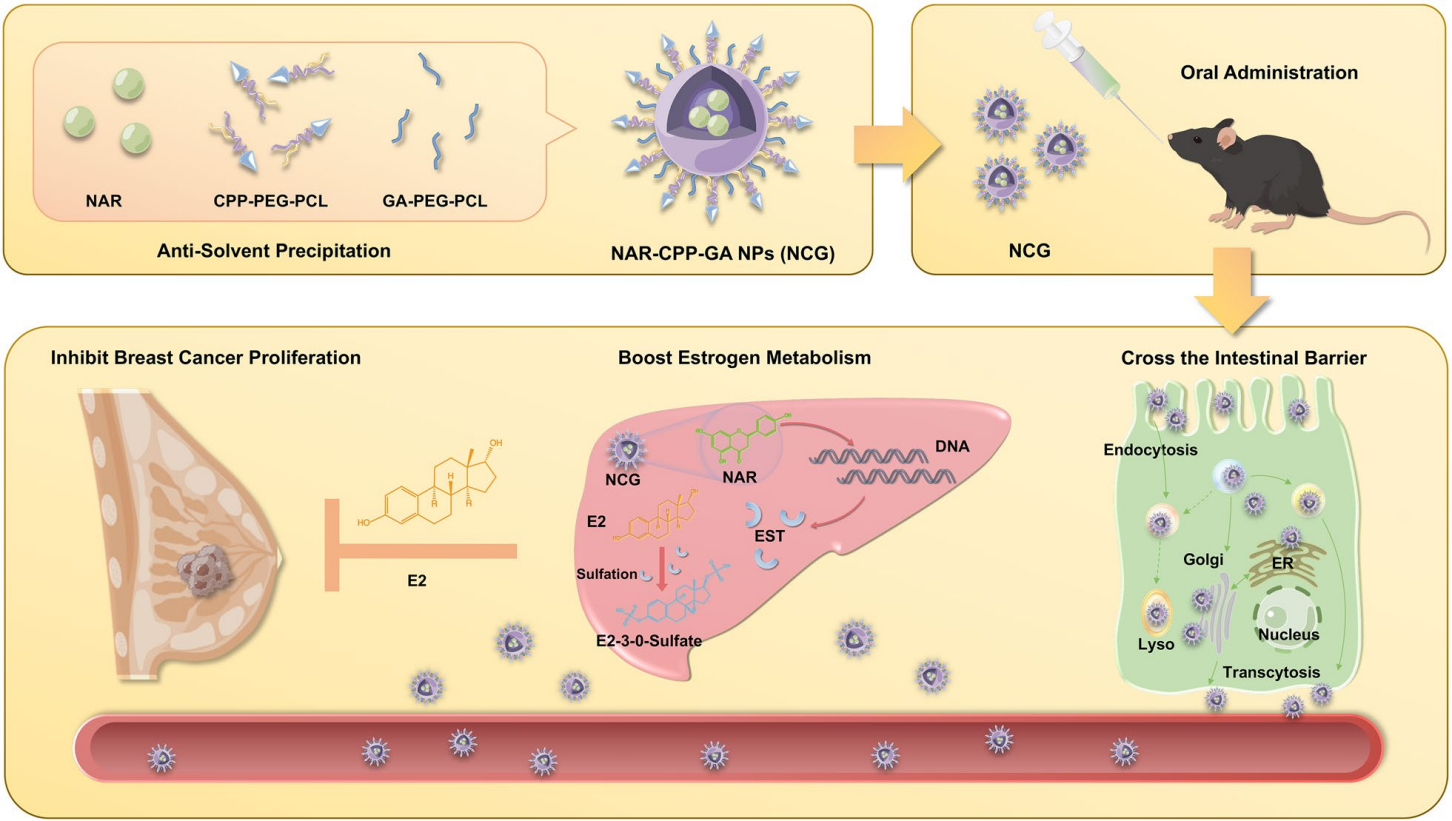

**Supplementary Information:**

The online version contains supplementary material available at 10.1186/s12951-024-02356-0.

## Introduction

Breast cancer is a leading cause of cancer-related death among women worldwide, accounting for approximately 25% of all new cancer cases and 15% of cancer-related deaths in women. Hormone receptor–positive (HR^+^) breast cancer represents approximately 70% of all cases and generally requires endocrine therapy for more than 5 years [1]. However, a number of patients develop resistance to endocrine therapy due to factors such as genetic and epigenetic alterations, signaling pathway crosstalk, and tumor heterogeneity [2, 3]. Notably, nearly 40% of patients with early estrogen receptor–positive (ER^+^) breast cancer experience recurrence within 15 years following adjuvant tamoxifen (TAM) therapy [4]. Moreover, the adverse effects associated with prolonged endocrine therapy also reduce patient quality of life and adherence to therapy. It is necessary and urgent to develop novel strategies for endocrine therapy with high efficiency and safety.

Endocrine therapy, targeting hormone receptors or estrogen synthesis [5], is the primary treatment for HR^+^ breast cancer [6]. Currently, different endocrine therapeutic strategies have been developed, such as the selective estrogen receptor modulators, aromatase inhibitors, and gonadotropin-releasing hormone agonist, as well as estrogen receptor degraders. In recent years, CKD4/6 inhibitors, such as abemaciclib and palbociclib, have been developed for salvage treatment of HR^+^ breast cancer patients [7]. With the comprehensive application of these strategies, the 5-year overall survival ratio of HR^+^ breast cancer has reached over 90% [8, 9]. However, even for the latest CDK4/6 inhibitors, it was reported that nearly 20% of patients exhibited primary resistance, and over 60% of patients appeared with secondary resistance after 40 months [10]. Meanwhile, the adverse effects associated with endocrine therapy, such as endometrial thickening, osteoporosis, and hepatoxicity, greatly limit clinical efficacy [11, 12]. Therefore, ongoing research is imperative to develop new drugs based on other endocrine-related signaling pathways and mechanisms.

Estrogen metabolism is crucial for maintaining dynamic hormone equilibrium [13]. Estradiol is the most biologically active form of estrogen, and is mainly metabolized by irreversible hydroxylations at either C-2, C-4, or C-16 through cytochrome P450 enzymes in the liver [14]. The metabolite 16α-hydroxyestrone has been shown to bind covalently to the ER and promote cell proliferation, and 2-hydroxyestrone and 4-hydroxyestrone were reported to contribute to potential genotoxic damage [13]. Conjugation is a subsequent step for increasing the water solubility of the hydroxylated metabolites, and thereby facilitating their excretion. The conjugating enzyme estrogen sulfotransferase (EST) catalyzes the transfer of the sulfate group from adenosine-5′-phosphate sulfate to any available hydroxyl group on the estrogen metabolites [15]. The EST-mediated sulfation process enhances estrogen’s water solubility, precludes binding of sulfated estrogen to the ER, and finally inactivates their activity. Previous studies also showed that EST induction in MCF-7 cells significantly inhibited cell proliferation and DNA synthesis [16]. EST disruption in female mice was reported to result in both local and systemic elevated estrogen [17–19]. Notably, EST is mainly expressed in hepatocytes [20–22]. Therefore, targeting hepatic EST expression has become a novel approach for breast cancer endocrine therapy.

At present, there are a limited number of drugs targeting hepatic EST to downregulate estrogen for HR^+^ breast cancer treatment. We previously found that Si-Ni-San, a traditional Chinese medicine formula, was capable of promoting estrogen metabolism in the liver, and naringenin (NAR) has been identified as a potential bioactive compound targeting EST [23]. However, the poor solubility and bioavailability of NAR limits its efficacy [24]. In the past decades, nanoparticle-based drug delivery systems have emerged as a powerful tool for cancer therapy, such as photothermal therapy [25], sonodynamics [26, 27], and redox targeting strategies [28]. Nevertheless, endocrine drugs for breast cancer treatment usually require long-term medication, and thus oral drug administration is the preferred route due to its noninvasiveness, high compliance, and low cost. However, the biological obstacles in the gastrointestinal tract pose a significant challenge for orally administered nanodrugs, such as the highly acidic environment, the diverse digestive enzymes, and the intact intestinal epithelium. Intriguingly, polymeric nanoparticles are particularly well-suited for oral drug delivery strategies due to their remarkable stability, low toxicity, high biocompatibility, and ease of modification. Currently, the U.S. Food and Drug Administration has approved the use of poly (ethylene glycol) (PEG) and poly (epsilon caprolactone) (PCL) polymers in pharmaceuticals [29]. In addition, cell-penetrating peptides (CPPs) are concise peptides that increase cellular uptake of diverse molecular carriers, such as small molecules, nucleic acids, and proteins, and facilitate their transfer across biological membranes. The application of CPPs has sparked significant interest in the field of drug delivery, particularly due to their ability to surmount the intestinal barrier, thereby favoring drug transport [30]. A key advantage of CPPs lies in their ability to transport an array of cargo molecules without triggering considerable cytotoxicity or immune reactions. Furthermore, CPPs can boost the bioavailability of insoluble or poorly permeable drugs, overcoming the difficulties of conventional oral drug delivery [30, 31]. It was demonstrated that incorporating CPPs with insulin remarkably improved insulin oral absorption in diabetic rats, highlighting the significance of CPPs in developing oral nanomedicines [32]. In terms of liver targeting, asialoglycoprotein receptors (ASGPRs) are specific receptors expressed on the surface of hepatocytes, and galactose (GA) is considered as a specific ligand binding to ASGPRs [33]. The administration of GA-bound nanoparticles facilitates preferential binding to ASGPRs, leading to receptor-mediated endocytosis and subsequent drug release within hepatocytes. Existing evidence indicated that GA-bound selenium nanoparticles significantly enhanced the antitumor effect of doxorubicin in hepatocellular carcinoma [34]. Consequently, developing oral liver-targeting drugs by amalgamating CPPs and galactose with bioactive compounds is a promising treatment approach.

In this study, we employed PEG-PCL coated NAR to synthesize nanoparticles, and introduced CPPs and GA onto the nanoparticle surface to improve their intestinal barrier permeability and hepatic targeting ability. Our results demonstrated that NCG exhibited specific liver targeting, enhanced EST elevating, and estradiol decreasing activity. Furthermore, NCG presented significant cancer inhibition with high safety in HR^+^ breast cancer xenografts. Our findings highlight that targeting hepatic estrogen metabolism is a promising therapeutic strategy for HR^+^ breast cancer, with NCG as a candidate oral nanomedicine for endocrine therapy (see Graphical abstract).

## Results and discussions

### Preparation and characterization of NCG

**Fig. 1:**
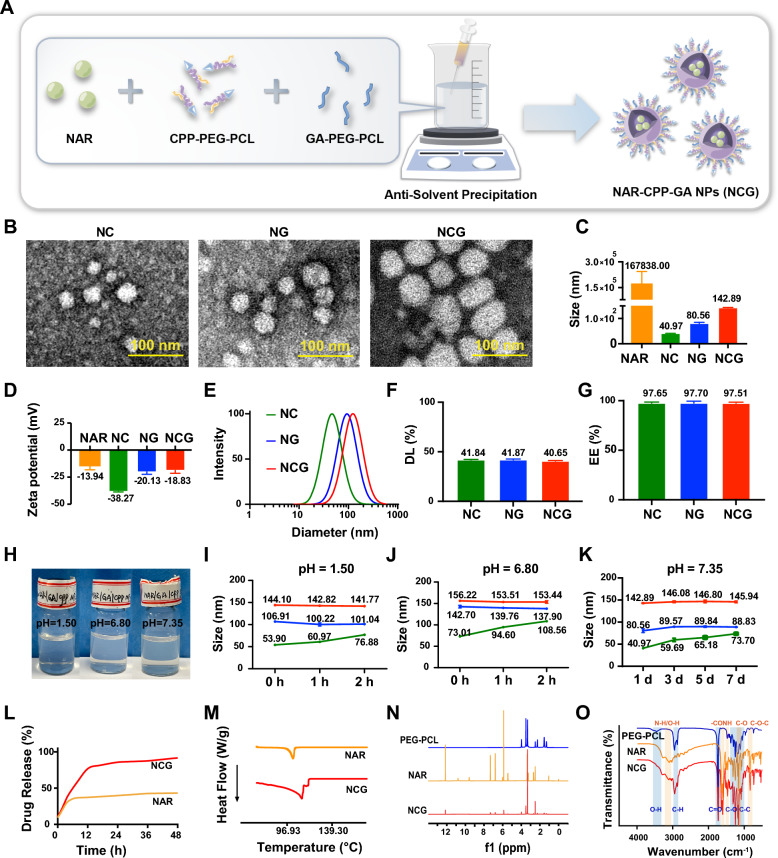
Characterization of NC, NG, and NCG. **A** The schematic diagram of NCG preparation process. **B** Transmission electron microscopy (TEM) images of NC, NG, and NCG. Scar bar: 100 nm. **C** Particle size of NAR, NC, NG, and NCG (n = 3). **D** Zeta potential of NAR, NC, NG, and NCG (n = 3). **E** Particles size distributions of NC, NG, and NCG. **F** Drug loading rate of NC, NG, and NCG (n = 3). **G** Encapsulation efficiency of NC, NG, and NCG (n = 3). **H** Visual graph of NCG stability at different pH values (n = 3). **I** Stability of NC, NG, and NCG in pH 1.50 environment. **J** Stability of NC, NG, and NCG in pH 6.80 environment. **K** Stability of NC, NG, and NCG in pH 7.35 environment. **L** In vitro drug release assay. The nanoparticles were exposed to simulated gastric juice with a pH of 1.50 for 2 h, simulated intestinal fluid with a pH of 6.80 for 2 h, and pH 7.35 phosphate-buffered saline (PBS) for 44 h to approximate blood pH. **M** DSC curves of NAR and NCG. **N** ^1^H NMR spectra of NAR, PEG-PCL, and NCG in CDCl3. **O** FT-IR spectra of NAR, PEG-PCL, and NCG

Our previous study reported that NAR in Si-Ni-San formula could inhibit breast cancer growth and metastasis by triggering hepatic EST expression. However, the liver-targeting ability and bioavailibity of NAR is low and limits its druggability. To overcome these challenges, several nanoparticles were assembled using NAR with Galactose-Polyethylene glycol (Mw: 6000) -Polycaprolactone (Mw: 2000) (GA-PEG-PCL) or Cell penetrating peptides (RRRRRRRRRRRR) -Polyethylene glycol (Mw: 6000) -Polycaprolactone (Mw: 2000) (CPP-PEG-PCL) according to previous literature methods (Fig. 1A) [35]. Additional file 1: Fig. S1A, B present the ^1^HNMR spectra of GA-PEG-PCL and CPP-PEG-PCL, respectively, indicating the successful synthesis of both compounds. The different self-assembling of NAR with PEG-PCL, GA-PEG-PCL, or CPP-PEG-PCL was called NN (NAR-PEG-PCL), NG (NAR-GA-PEG-PCL), NC (NAR-CPP-PEG-PCL), and NCG (NAR-CPP-GA-PEG-PCL), respectively. The nanoparticle with fluorescence labeling is referred as Cy5-NCG. Upon examination under an electron microscope, NC, NG, and NCG (Fig. 1B) were found to be spherical in shape, with particle sizes of 40.97 ± 0.57 nm, 80.56 ± 4.40 nm, and 142.89 ± 0.94 nm, respectively (Fig. 1C). Surface charge measurements of NAR were − 13.94 ± 4.30 mV, while those of NC, NG, and NCG were − 38.27 ± 0.42 mV, − 20.13 ± 2.24 mV, and − 18.83 ± 2.61 mV, respectively (Fig. 1D). The particle size of Cy5-NCG was 111.72 ± 4.15 nm and the surface potential was − 10.10 ± 0.74 mV (Additional file 1: Fig. S1C). As shown in Fig. 1E, the polydispersity index of NC, NG, and NCG were 0.14 ± 0.01, 0.16 ± 0.01, and 0.17 ± 0.01, indicating that these nanoparticles presented uniform distribution. Furthermore, the drug-loading and encapsulation efficiencies of NCG reached 40.65% and 97.51%, respectively, similar to those of NC and NG (Fig. 1F, G), highlighting the excellent drug-loading capacity of the synthesized nanoparticles.

To assess the stability and acid resistance, NCG was placed in transparent bottles containing simulated gastric fluid, intestinal fluid, or PBS to monitor whether precipitation occurred. The results showed that NCG remained stable in both simulated gastric fluid and intestinal fluid, with no precipitation occurring within 2 h (Fig. 1H). Notably, NCG exhibited minimal changes in size within 2 h in either simulated gastric fluid (pH 1.50) or intestinal fluid (pH 6.80). When the pH value was set at 7.35 to imitate the blood microenvironment, no significant change in size of the NCG nanoparticles was found within 7 days (Fig. 1I–K). In addition, NCG in the transparent vials showed no precipitation after 30 days at pH 7.35 (Additional file 1: Fig. S1D), indicating the high stability of NCG under storage conditions. For drug release experiments, to better mimic the oral drug administration route in humans, the external medium was set as simulated gastric fluid for the first 2 h, and followed by intestinal fluid from 2 to 4 h, and PBS from 4 to 48 h. The results showed that NCG presented a faster drug release capacity than NAR. The drug released by NCG reached as high as 91.66% at 48 h, but was only 43.20% in the NAR group (Fig. 1L). In differential scanning calorimetry analysis, a single endothermic peak was detected at 111 °C when NCG nanoparticles were analyzed, consistent with the peak of NAR (Fig. 1M), suggesting the properties of NAR within NCG are not impacted by the antisolvent precipitation process.

For structure validation assays, ^1^H-NMR spectroscopy was utilized to characterize the PEG-PCL polymer, NAR, and NCG. As shown in Fig. 1N, the results revealed peaks at 3.34 ppm and 3.61 ppm that matched the methylene and methyl protons of PEG, respectively. Compared to the proton spectra of PEG-PCL and NAR, NCG did not show any new functional groups, indicating that NAR and PEG-PCL formed a simple encapsulation rather than a chemical reaction. FT-IR spectroscopy is one of the most effective techniques for assessing the chemical stability of encapsulated drugs in nanoparticles. The FT-IR spectra shown in Fig. 1O confirmed the successful association between NAR and PEG-PCL. In the PEG-PCL infrared analysis, the peaks at 2869 cm^−1^ and 2947 cm^−1^ were consistent with C–H stretching, peaks appearing in the range of 1090–1300 cm^−1^ were consistent with C–C and C–O, and peaks at 1728 cm^−1^ and 3443 cm^−1^ were consistent with C=O and O–H, respectively. Further analysis identified peaks in the range of 1085–1150 cm^−1^, which were attributed to the ether bands of PEG. These unique bands of free PEG-PCL were present in NCG. In the NAR infrared analysis, the band appearing at approximately 3284 cm^−1^ was due to N–H/O–H stretching vibrations, and the bands at approximately 1635 cm^−1^ and 1464 cm^−1^ were attributed to –CONH amide I and CH_2_ bending vibrations, respectively. The infrared bands at approximately 1155 and 834 cm^−1^ were attributed to C–O and C–O–C stretching vibrations. These unique bands of free NAR were also found in NCG, indicating the chemical stability of NAR and PEG-PCL within NCG. Overall, these findings suggested the successful preparation of NCG.

### NCG exhibits specific liver targeting and increased intestinal barrier permeability in vitro

**Fig. 2:**
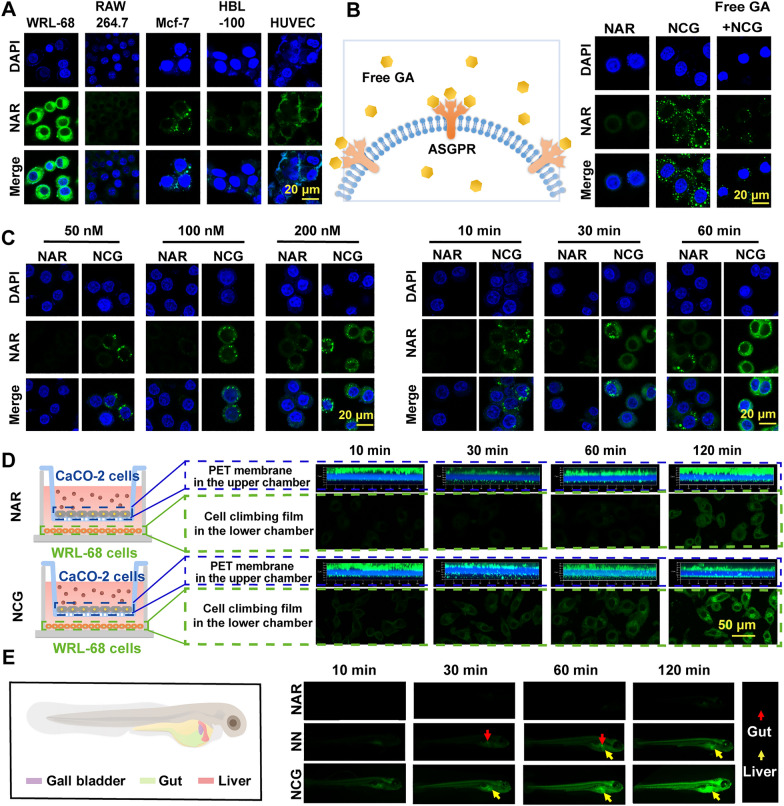
In vitro assessment of the liver targeting and intestinal barrier penetration ability of NCG. **A** Examination of NAR and NCG uptake by different cells, scar bar: 20 μm; **B** evaluation of the impact of ASGPR on NCG liver targeting, scar bar: 20 μm; **C** evaluation of NAR and NCG uptake in WRL-68 cells at 30 min and assessment of NAR and NCG uptake in WRL-68 cells at 10 min, 30 min, and 60 min, scar bar: 20 μm; **D** assessment of the capability of NAR and NCG penetrating the Caco-2 cell layer and the quantity absorbed by WRL-68 cells in the lower chamber, scar bar: 50 μm; **E** Zebrafish organ pattern diagram, and the uptake and distribution of NAR, NAR NPs, and NCG uptake by zebrafish; the green fluorescence indicates NAR

To explore the liver-targeting capability of NCG, it was incubated with various cell types. Under the same concentration and time conditions, NCG displayed the highest uptake in WRL-68 liver cells, which significantly exceeding the uptake of other cell types and thus indicated its liver-targeting ability in vitro. Additionally, minimal NAR fluorescence was detected in RAW 264.7 cells, indicating that NCG was rarely engulfed by macrophages, which may contribute to the avoidance of immune rejection (Fig. 2A). Notably, when NCG was incubated with ASGPR-saturated liver cells, the cellular uptake of NCG was severely reduced, suggesting that the liver targeting ability of NCG was facilitated by the binding of GA and ASGPR (Fig. 2B). To further assess the differences in cellular uptake between NAR and NCG, WRL-68 liver cells were individually incubated with NAR and NCG. The findings revealed that NAR fluorescence in WRL-68 cells was less intense and dimmer than NCG fluorescence, indicating that NCG was more easily internalized by WRL-68 cells, and exhibited dose-dependent uptake. Similarly, NCG treatment led to a faster emergence of NAR fluorescence in WRL-68 cells as the time increased, suggesting that NCG demonstrated time-dependent uptake compared to NAR (Fig. 2C).

In addition, to evaluate the ability of NCG to cross the intestinal barrier, we used a Transwell co-culture system to inoculate Caco-2 cells onto the PET membrane in the upper chamber of the Transwell system. Samples with epithelial resistance (TEER) > 180 Ω/cm^2^ were considered suitable for simulating the intestinal barrier. After 120 min, only a small quantity of NAR fluorescence penetrated the dense Caco-2 cell layer, and 70.22% of the initial NAR concentration remained in the upper chamber (Additional file 1: Fig. S2A). This phenomenon could be attributed to the challenging cellular uptake of NAR or the necessity for a longer incubation time. Conversely, in the NCG group, the green fluorescence of NAR penetrating through the Caco-2 cells increased remarkably over time. After 120 min, only 28.17% of the initial naringin concentration persisted in the upper chamber (Additional file 1: Fig. S2A). This result suggests that the CPP penetration peptide facilitated NCG crossing the intestinal barrier (Fig. 2D). To determine whether the NCG that penetrated the Caco-2 cells remained intact and was internalized again by WRL-68 cells, we seeded WRL-68 cells on a slide in the lower chamber. The findings demonstrated that the NCG that passed through the Caco-2 cell layer was internalized by WRL-68 cells in a time-dependent manner. As shown in Fig. 2D, the penetrated NCG surrounded the WRL-68 cells at 10 min and had not yet entered into the cells. Over time, NCG was progressively taken up by WRL-68 cells, and the cellular fluorescence intensity steadily increased. The quantitative analysis of the cellular fluorescence is shown in Additional file 1: Fig. S2B.

To further validate the intestinal barrier permeability and liver-targeting capabilities of NCG, different drugs were co-incubated with zebrafish. Zebrafish mainly absorb external substances through their mouth, naturally serving as a real-time visualization tool for drug distribution via oral administration. Referring to the position of the zebrafish organs (Fig. 2E), the results indicated that the fluorescence of the NAR group was remarkably weak, due to its poor water solubility and limited digestion. In contrast, NP and NCG exhibited varying degrees of uptake, and presenting as a time-dependent pattern. Notably, NCG appeared earlier in the zebrafish liver tissue, and the fluorescence in the liver tissue was more intense than in the NP group. This result indicated the notable liver-targeting and intestinal barrier penetration ability of NCG in vivo.

### NCG efficiently releases in hepatic cells following caveolin-mediated endocytosis

**Fig. 3:**
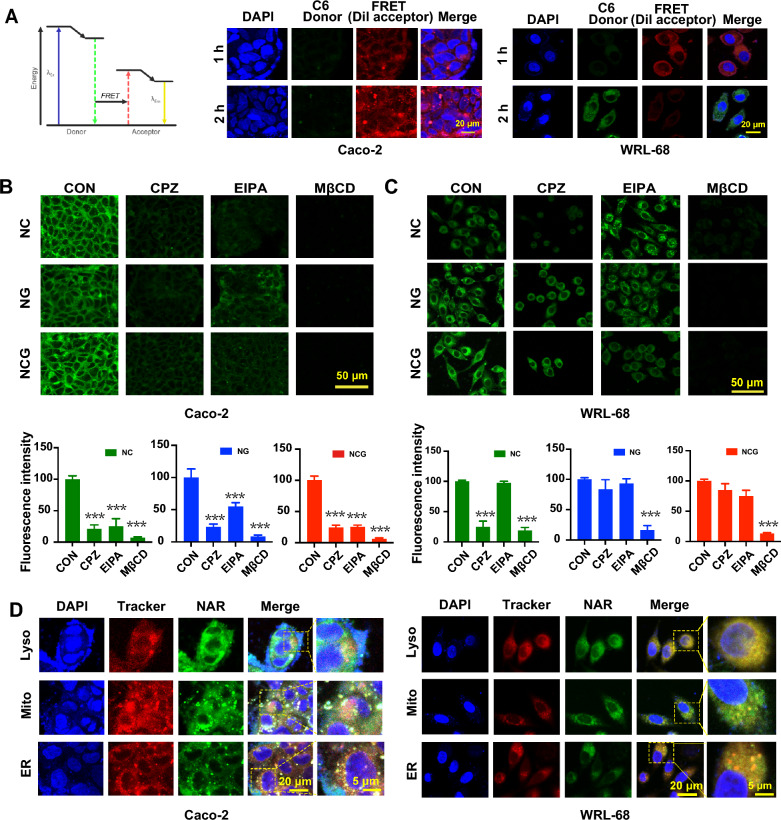
Intracellular integrity, uptake mechanisms and distribution of NCG. **A** Schematic diagram of FRET technology. Blue fluorescence corresponds to DAPI, green fluorescence signifies donor C6, and red fluorescence denotes acceptor DiI, scar bar: 20 μm. **B**, **C** The effects of various endocytosis inhibitors on the uptake of NC, NG, and NCG by Caco-2 cells and WRL-68 cells are examined. Statistical analysis of fluorescence intensity for NC, NG, and NCG groups in Caco-2 cells and WRL-68 cells under different endocytosis inhibitors is provided (n = 6). Data are presented as mean ± SD, ***p < *0.001* *compared to the Control group*. Scale bar: 50 μm. **D** The co-localization of NCG with lysosomes (Lyso), endoplasmic reticulum (ER), and mitochondria (Mito) in Caco-2 and WRL-68 was visualized using appropriate tracking dyes. Scale bar: 20 μm

Based on the above results, NCG nanoparticles were efficiently transported through the intestinal barrier and subsequently digested by liver cells. It was essential to determine the integrity, uptake mechanism, and intracellular distribution of NCG in Caco-2 and WRL-68 cells. Förster resonance energy transfer (FRET) technology was utilized to examine the integrity of NCG in Caco-2 and WRL-68 cells. Fluorophores Coumarin 6 (C6) and 1,1′-dioctadecyl-3,3,3′,3′-tetramethylindocarbocyanine perchlorate (DiI) were encapsulated within the nanoparticles. When the distance between the two fluorophores was less than 10 nm, the emission of C6 activated the emission of DiI, resulting in red fluorescence and indicating the integrity of the NCG nanoparticles. Conversely, when the distance exceeded 10 nm, the green fluorescence emitted by C6 could not activate DiI, leading to only the green fluorescence of C6, which suggested the disintegration of the nanoparticle (Fig. 3A). FRET results indicated that NCG maintained its integrity within the Caco-2 cells for 2 h, which was adequate for crossing the Caco-2 cell layer. In WRL-68 cells, NCG remained intact for the first hour, but by approximately 2 h, the fluorescence pairs within the nanoparticles were completely released.

To elucidate the uptake mechanism of NCG in Caco-2 and WRL-68 cells, NAR and NCG were co-incubated with WRL-68 and Caco-2 cells and treated with different endocytic pathway inhibitors, including chlorpromazine (clathrin-mediated endocytosis inhibitor), 5-(*N*-ethyl-*N*-isopropyl)-amiloride (macropinocytosis inhibitor), and methyl-β-cyclodextrin (caveolin-mediated endocytosis inhibitor). The findings demonstrated that NC entered into WRL-68 cells primarily through clathrin-mediated endocytosis and caveolin-mediated endocytosis, but was digested by Caco-2 cells via all three endocytic pathways. NG entered into WRL-68 cells primarily through caveolin-mediated endocytosis, but were taken up by Caco-2 cells mainly through clathrin-mediated endocytosis and caveolin-mediated endocytosis. NCG entered into WRL-68 cells mainly through caveolin-mediated endocytosis, and all three endocytic pathways were involved in its entry into Caco-2 cells. The relative values of intracellular fluorescence were quantified as shown in Fig. 3B, C.

Different endocytic pathways also determined the fate of the nanoparticles after cellular entry. Co-localization with subcellular organelles revealed that, in Caco-2 cells, NCG was primarily distributed in the endoplasmic reticulum and seldomly accumulated in lysosomes and mitochondria. Minimal lysosomal accumulation indicated that NCG traversed Caco-2 cells without degradation (Fig. 3D). In WRL-68 cells, NCG was mainly distributed in the endoplasmic reticulum and lysosomes, with minimal presence in mitochondria, suggesting that NCG could regulate cellular signaling via the Golgi-endoplasmic reticulum pathway. Additionally, NCG nanoparticles were degraded by lysosomes in WRL-68 cells, indicating a high safety. Minimal mitochondrial accumulation indicated that NCG had limited impact on mitochondrial respiration, indicating its low cytotoxicity.

### NCG significantly activates EST expression and promotes estradiol metabolism in hepatocytes

**Fig. 4:**
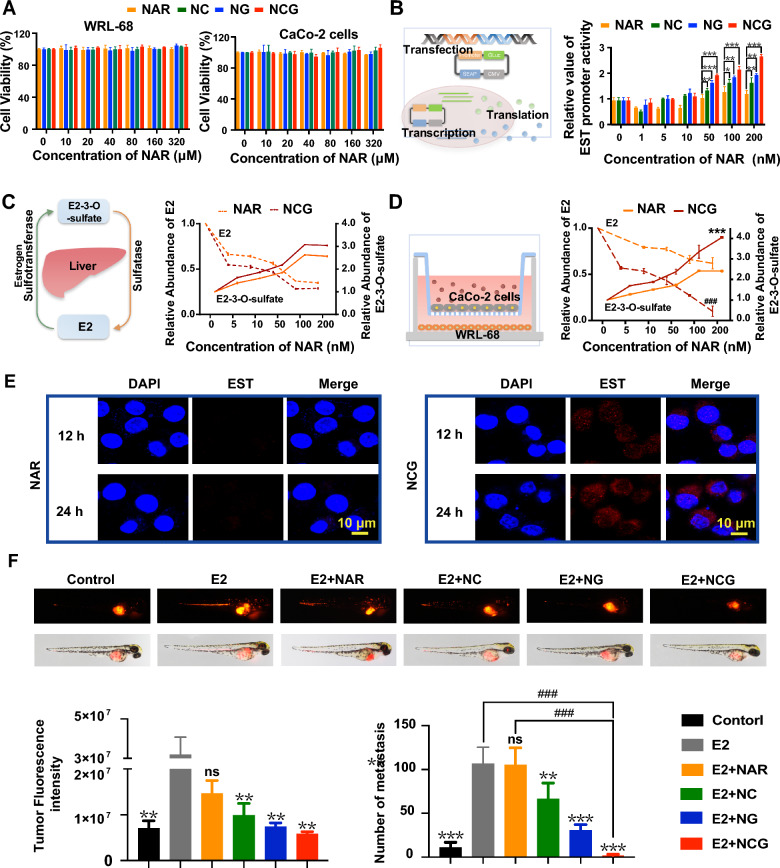
Effects of NCG on the viability, EST expression and estradiol metabolism in hepatocytes. **A** Evaluation of cytotoxicity in WRL-68 and Caco-2 cells for various NAR formulations (n = 3). **B** Assessment of the ability of multiple NAR formulations to activate EST genes at 24 h using the dual luciferase reporter gene assay (n = 3). Data are presented as mean ± SD, *p < *0.05*, **p < *0.01*, ***p *< 0.001* indicates a comparison between the two groups. **C** Impact of NAR and NCG on estradiol metabolism in WRL-68 cells. **D** Influence of NAR and NCG on estrogen metabolism and **E** expression of EST enzyme in WRL-68 cells seeded at the lower chamber of a Transwell system after 24 h (n = 3). Data are presented as mean ± SD, ***p < *0.001*, ^###^*p* < *0.001* compared to the NAR group. Scar bar: 10 μm*;* **F** modulation of breast cancer growth and metastasis in a zebrafish xenograft model by NAR, NC, NG, and NCG through regulation of estradiol (40 μmol/L) metabolism. Statistical analysis of tumor fluorescence intensity and metastatic foci count in zebrafish (n = 6). Data are presented as mean ± SD, ns: *p* > *0.05*, **p < *0.01*, ***p < *0.001* compared to the E2 group; ^###^*p* < *0.001* indicates a comparison between the two groups

To test the cytotoxicity effects of NCG, we co-cultured NAR, NC, NG, and NCG with WRL-68 and Caco-2 cells. CCK8 assay results revealed that NAR, NC, NG, and NCG exhibited no significant toxicity towards WRL-68 or Caco-2 cells at concentrations as high as 320 μmol/L (Fig. 4A). Furthermore, we assessed the cytotoxicity of these drugs on other cell types, including RAW264.7 (Additional file 1: Fig. S3A), HBL-100 (Additional file 1: Fig. S3B), HUVEC (Additional file 1: Fig. S3C), and MCF-7 (Additional file 1: Fig. S3D) cells. The findings indicated that NAR displayed only mild toxicity to MCF-7 cells at a concentration of 320 μmol/L, with a cell survival rate of 83.44% and no statistically significant difference. The other drugs showed minimal toxic effects on all cell types.

EST is mainly responsible for converting estrogen into sulfated metabolites that cannot bind with the ER, and therefore inactivate the estrogen signaling pathway [15, 36]. It was found that the highest total activity of EST in guinea pigs is in the liver, where estrogen metabolism mainly occurs [37]. Increased EST expression in the liver has been found to significantly reduce estrogen levels in the liver, blood, and tumors, accompanied by simultaneously elevated sulfated metabolites [38]. Therefore, we next investigated the impact of NCG on enhancing EST gene expression in hepatocytes, and a dual-luciferase reporter gene assay was utilized (Fig. 4B). After drug treatment of WRL-68 cells for 4 h, minimal influence on EST gene expression was observed (Additional file 1: Fig. S3E). At 12 h, EST gene expression presented a dose-dependent increase in all groups, with significant changes of NCG group over 50 nmol/L and NG group at 200 nmol/L (Additional file 1: Fig. S3F). At 24 h, the NC, NG, and NCG groups all significantly promoted EST gene expression compared to the NAR group in the concentration range of 50–200 nmol/L (Fig. 4B). To validate the promoting effects of NCG on estradiol metabolism, mass spectrometry was applied to measure the levels of E2 and E2-3-*O*-sulfate metabolized by WRL-68. WRL-68 cells were treated with 40 μmol/L estradiol added to the culture medium, followed by administration of NAR and NCG. The results (Fig. 4C) showed that both NAR and NCG resulted in a significant reduction of E2 content and a notable increase in the E2-3-*O*-sulfate level in a dose-dependent manner at 24 h. However, no significant difference was observed between NAR and NCG in facilitating E2 metabolism, which might be attributed to the direct drug administration route. Therefore, a Transwell co-culture system was established by loading Caco-2 cells in the upper chamber and WRL-68 cells in the lower chamber, and NAR and NCG was subsequently administrated to Caco-2 cells. The results showed that the promoting effects of NAR on estradiol metabolism significantly decreased, while NCG significantly enhanced the transformation from E2 to E2-3-*O*-sulfate (Fig. 4D). This finding suggested that the intestinal barrier-crossing ability of NAR was poor, but NCG successfully traversed the Caco-2 cell layer and was taken up by hepatocytes to promote estradiol metabolism. Moreover, the expression level of EST in WRL-68 hepatocytes in the lower chamber was analyzed. Immunofluorescence results revealed that NAR did not affect the EST level, but NCG led to significantly enhanced expression (Fig. 4E), which was attributed to the upregulation of E2-3-*O*-sulfate.

To validate the anti-cancer effects of NCG in vivo, the ER^+^ breast cancer cell line E0771 was used to establish a zebrafish xenotransplantation model. Estradiol and different drugs were subsequently added to monitor breast cancer growth and metastasis. The findings revealed that the positive drug estradiol significantly enhanced breast cancer growth and metastasis, which was significantly blocked by NC, NG, and NCG (Fig. 4F). The inhibitory effects of NCG were the highest, corresponding to the highest absorption.

### NCG demonstrates hepatic targeting and enterohepatic circulation in vivo

**Fig. 5:**
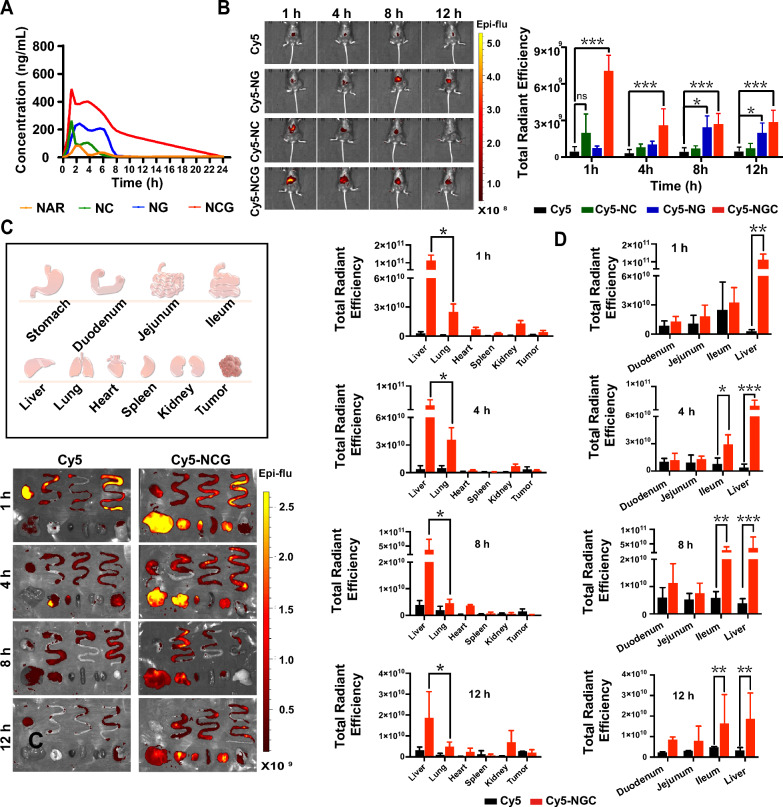
In vivo pharmacokinetics and distribution of NCG. **A** Pharmacokinetic curves of different NAR formulations are shown after oral administration at a dosage of 6.9 mg/kg in rats (n = 6). **B** Representative images and a statistical analysis of fluorescence intensity for the in vivo distribution of various nanoparticle formulations (n = 3). Data are presented as mean ± SD, ns: *p* > *0.05*, *p < *0.05*, ***p < *0.001* indicates a comparison between the two groups*.* **C** Representative distribution of Cy5 in major organs and a statistical analysis of fluorescence intensity (*n* = 3). Data are presented as mean ± SD, *p < *0.05* indicates a comparison between the two groups. **D** Statistical analysis of fluorescence intensity in duodenum, jejunum, ileum, and liver (n = 3). Data are expressed as mean ± SD, *p < *0.05*, **p < *0.01*, ***p *< 0.001* indicates a comparison between the two groups

Pharmacokinetic studies (Fig. 5A) were carried out to examine the in vivo metabolism of various NAR formulations. The plasma pharmacokinetic parameters (Additional file 1: Table S1) showed that the T_1/2_ of NCG (5.06 ± 1.52 h) was significantly longer than that of free NAR (2.74 ± 0.34 h), NC (4.51 ± 1.20 h), and NG (4.99 ± 1.21 h), suggesting that NCG possessed an extended blood circulation half-life. Furthermore, NCG achieved a higher C_max_ (507.04 ± 13.28 μg/L) than free NAR (87.99 ± 14.81 μg/L). The AUC_0−t_ was also higher in the NCG group (3393.19 ± 245.41 μg h/L) than in the NAR group (360.98 ± 144.81 μg h/L). The bioavailability of NC, NG, and NCG significantly increased by 1.82-, 3.97-, and 9.39-fold, respectively, when compared with free NAR. Moreover, NAR, NC, NG, and NCG exhibited bimodal pharmacokinetics, which is generally attributed to the occurrence of enterohepatic circulation.

Cy5 (cyanine 5) is a cyanine fluorescent dye characterized by far-red fluorescence emission, long wavelengths, and strong penetration properties, making it a common choice for in vivo imaging in small animals. NC, NG, and NCG were labeled with Cy5 and subsequently given to mice via oral administration to monitor drug distribution at 1 h, 4 h, 8 h, and 12 h. The results demonstrated that the Cy5 group exhibited the weakest fluorescence intensity and the lowest Cy5 accumulation in the liver. Fluorescence of Cy5-NC in the liver was detected at 1 h after oral administration; however, it gradually decreased over time and was completely metabolized at approximately 12 h. The Cy5-NG group fluorescence peaked at 8 h, indicating that only a small proportion of Cy5-NG reached the liver before 4 h after oral administration. Notably, the Cy5-NCG group displayed the highest liver fluorescence as early as 1 h after oral administration, with the fluorescence persisting for 12 h (Fig. 5B). These findings suggested that NCG rapidly crossed the intestinal barrier following oral administration and remained in the liver for an extended duration, therefore allowing sufficient time for drug release and activity. Although previous research has sought to encapsulate NAR within liposomes or a solid dispersion to enhance its dissolution and absorption, the liver-targeting strategy and the convenience of the oral administration route is usually neglected, which is important and necessary for the long-term treatment of endocrine drugs [39]. By leveraging nanotechnology, the solubility, stability, intestinal barrier penetrability, and liver-targeting specificity of NAR was significantly improved.

In light of the aforementioned insights, a closer look into organ-specific distribution is worth to be explored. Therefore, the mouse organs were extracted at different time points after oral administration and analyzed using an in vivo imaging system (Fig. 5C). The results revealed that the Cy5 group’s fluorescence was primarily localized in the stomach and intestines, with minor presence in the heart, liver, spleen, lung, and kidney, as well as in the tumor. This phenomenon suggested that the majority of Cy5 failed to penetrate the intestinal barrier after oral administration and was excreted through feces within 4 h. By contrast, Cy5-NCG was predominantly concentrated in organs such as the liver and lung, and was maintained in the body as long as 12 h. Intriguingly, the fluorescence of Cy5-NCG was repeatedly detected in the liver, duodenum, jejunum, and ileum (Fig. 5D), a phenomenon akin to previous enterohepatic circulation reports [40]. This finding implied that NCG may possess an enterohepatic circulation ability, which contributed to the prolonged drug action time and enhanced drug bioavailability.

### NCG inhibits HR^+^ breast cancer growth by promoting hepatic EST expression

**Fig. 6:**
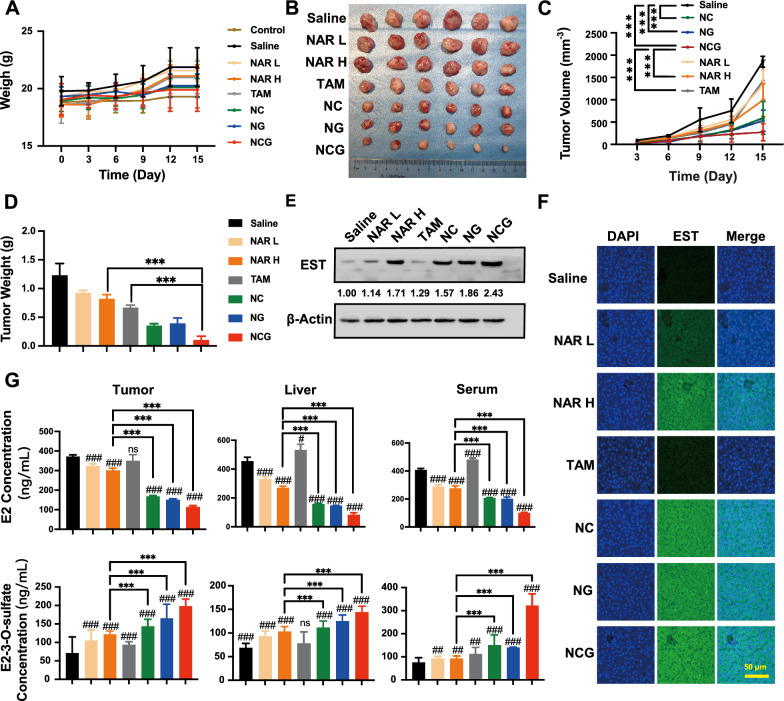
Effects of NCG on HR^+^ breast cancer growth and estradiol metabolism in vivo. **A** Body weight curves in mice across various treatment groups (n = 6). **B** Representative tumor images in mice. **C** Breast cancer volume curves for different groups (n = 6), Data are presented as mean ± SD, ***p < *0.001* indicates a comparison between the two groups. **D** Breast cancer weights in different groups (n = 6) at treatment completion, Data are presented as mean ± SD, ***p < *0.001* indicates a comparison between the two groups. Expression levels of the EST enzyme in mouse liver tissue, as assessed by Western blotting (**E**) and immunofluorescence (**F**) across groups. Scar bar: 50 μm. **G** Effects of various groups on estradiol and estradiol-3-*O*-sulfate concentrations in liver, serum, and tumors (n = 6). Data are presented as mean ± SD, ***p < *0.001* indicates a comparison between the two groups; ns: *p* > *0.05*, ^#^*p* < *0.05*, ^##^*p* < *0.01*, ^###^*p* < *0.001* compared to the Control group

To further evaluate the inhibition effects of NCG on ER^+^ breast cancer growth, mouse orthotropic breast cancer xenografts were established by using E0771 cells, and different drugs or saline were given to each group via oral administration. The results showed little significant difference in body weight between the control group and the drug-treated groups (Fig. 6A). Furthermore, it was shown that the free NAR dose-dependently inhibited breast cancer growth. However, the tumor inhibition ratio of the high dosage of NAR reached only 33.33%, which was still low when compared to 46.34% in the positive drug TAM group. Notably, the NG and NC groups demonstrated higher tumor suppression effects, with inhibition rates of 71.54% and 68.29%, respectively, which were superior to that of TAM. The highest cancer inhibition effects were observed in NCG group, which significantly limited cancer growth by 91.86%, and was remarkably superior to the positive drug TAM (Fig. 6B, C). A similar phenomenon was also achieved in the tumor weight comparison (Fig. 6D). Based on in vitro results, the NCG-induced tumor inhibition might be attributed to a reduction of estradiol. Therefore, Western blot assays were used to detect the expression level of the EST enzyme in the liver of the mice in different groups. It was found that high-dosage NAR, NC, NG, and NCG significantly promoted EST expression in the liver, and NCG treatment led to an increase of 2.42-fold, which was the highest (Fig. 6E). The immunofluorescence results were also consistent with the findings (Fig. 6F and Additional file 1: Fig. S4), further confirming the promoting effects of NCG on EST expression in the liver. Mass spectrometry was used to detect the level of estradiol and its metabolites in the blood, tumor, and liver of mice to validate the regulatory effects of NCG on estrogen metabolism (Fig. 6G). Compared with the saline group, the estradiol level in the liver and serum of the TAM group increased significantly, which was related to the mechanism of action of TAM, which caused a competitive increase in the estradiol content. The administration of NAR significantly alters estrogen levels in the liver, serum, and tumors, although its reduction effect is weaker compared to NCG. This was mainly due to the poor water solubility and stability of free NAR, resulting in its low bioavailability. Compared with the free NAR group, the estradiol level in the NC, NG, and NCG groups was significantly reduced, accompanied by the increased content of the E2-3-*O*-sulfate metabolite in tumors, liver tissue, and serum. Taken together, these findings suggested that NCG efficiently inhibited HR^+^ breast cancer growth by promoting hepatic EST expression and decreasing the estradiol level in vivo.

### NCG shows good biocompatibility in vivo

**Fig. 7:**
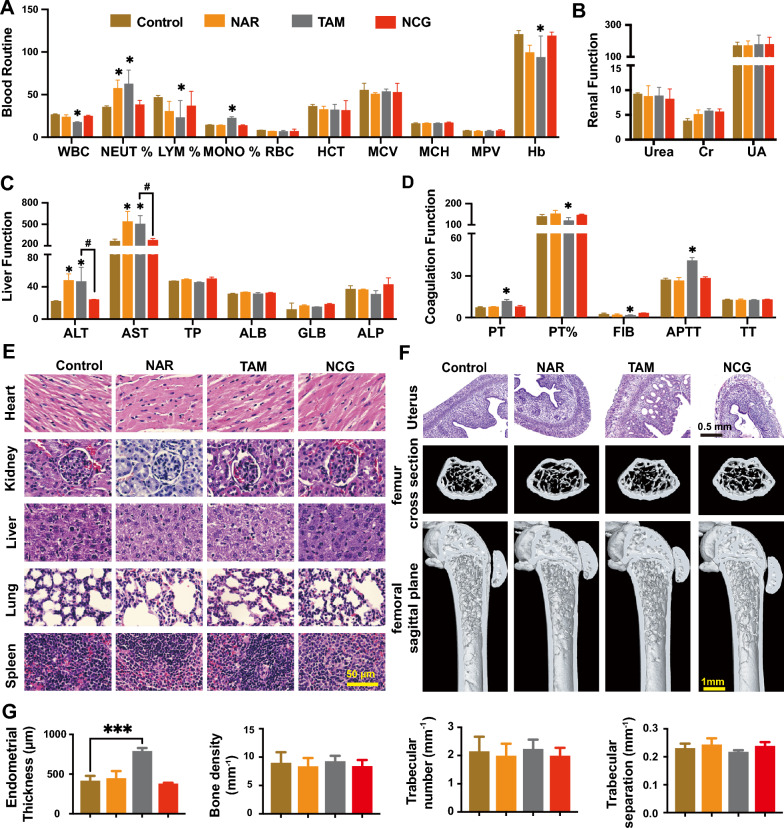
In vivo biocompatibility of NCG. **A** The blood routine parameters in different treated mice groups (n = 3). white blood cells (WBC), neutrophils ratio (NEUT%), lymphocyte ratio (LYM%), monocyte ratio (MONO%), red blood cells (RBC), hematocrit (HCT), mean corpuscular volume (MCV), mean corpuscular hemoglobin (MCH), mean platelet volume (MPV), and hemoglobin (Hb). Data are presented as mean ± SD, *p < *0.05* compared to the Control group. **B** The changes of urea, creatinine (Cr), and uric acid (UA) in different treated mice group (n = 3); **C** the levels of alanine aminotransferase (ALT), aspartate aminotransferase (AST), total protein (TP), albumin (ALB), globulin (GLB), alkaline phosphatase (ALP) levels in different treated mice groups (n = 3). Data are presented as mean ± SD, *p < *0.05* compared to the Control group; ^#^*p* < *0.05* indicates a comparison between the two groups. **D** Coagulation function in mice across various treatment groups (n = 3). Prothrombin time (PT), prothrombin time ratio (PT%), fibrinogen (FIB), active partial thromboplastin time (APTT), thrombin time (TT). Data are presented as mean ± SD, *p < *0.05* compared to the Control group. **E** Representative pathological images of major organs in mice from different treatment groups. Scar bar: 50 μm. **F** Representative pathological images of uterine sections and endometrial thickness statistics for mice in distinct treatment groups (scar bar: 0.5 mm), and representative micro-CT images (scar bar: 1 mm) of femurs in mice from various treatment groups, accompanied by bone density, trabecular bone number, and trabecular separation statistics (**G**) (n = 3). Data are presented as mean ± SD, ***p < *0.001* compared to the Control group

Endocrine drugs prescribed to breast cancer patients usually require long term administration for 5 to 10 years, and thus bring severe side effects [41]. For example, TAM usually causes coagulation disorders and endometrial thickness, while aromatase inhibitors may lead to osteoporosis [42, 43]. These side effects can potentially reduce patient compliance to their medication and ultimately influence the overall effectiveness of endocrine therapy. Here, to test the biocompatibility of NCG, mouse blood was collected to detect the changes in blood cell counts, coagulation function, and liver and kidney function. It was found that the percentage of neutrophils, alanine aminotransferase, and aspartate aminotransferase in the NAR group significantly increased (Fig. 7A–C), which may be due to the rapid growth of the tumors in mice, causing a immune system abnormalities and liver damage. The phenomenon was similar in the TAM group. Notably, the prothrombin time and activated partial thromboplastin time in the TAM group were significantly prolonged, and the level of plasma fibrinogen was reduced, indicating that TAM may result in coagulation disorders, which may be related to the inhibition of platelet aggregation by TAM [44–46] (Fig. 7D). However, after 14 days of oral NCG treatment, no abnormalities in routine blood, coagulation, liver, or kidney function occurred in mice, indicating that NCG has good biocompatibility. The phenomenon might be attributed to the low cytotoxicity of NAR, which did not inhibit the proliferation of normal hepatic and colon cells when its concentration reached as high as 320 μmol/L. Polycaprolactone-polyethylene glycol copolymer in the NCG formula was also approved by the United States Food and Drug Administration due to its exceptional biocompatibility and biodegradability. GA has also been applied in clinic use as a bioactive ingredient for hepatoprotection, inflammation inhibition, and immunological disorders. Clinical trials of peptide-conjugated drugs are underway and show tremendous potential for cancer therapy. All these factors contribute to the high safety of NCG in vivo.

Pathological examination of the main organs of the mice was also performed. Hematoxylin–eosin staining showed that minimal organ damage was caused by any treatment when compared with the control group (Fig. 7E). Since endometrial thickening is a common side effect of TAM caused by the elevated estradiol, the mouse uteri were collected to evaluate the endometrial thickness of mice in different treatment groups (Fig. 7F). The results showed that, compared with the control group, the endometrial thickness of TAM-treated mice significantly increased (Fig. 7G), while there were no significant changes in the other treatment groups. It is well known that decreased estradiol can result in osteoporosis. Micro-CT imaging of the femur was subsequently performed to evaluate whether the mice developed osteoporosis. As shown in Fig. 7F, G, the bone density, trabecular number, and trabecular separation of the mouse femur in NCG group was comparable between the control, NAR, and TAM groups, without any statistical difference. These results indicated that although NCG promoted estradiol inactivation, it did not induce osteoporosis. Previous reports also demonstrated that NAR inhibited osteoporosis induced by ovariectomy [47], but the molecular mechanisms remain to be further clarified. Overall, NCG is well tolerated in vivo and deserves further clinical research.

## Conclusions

This study provides an alternative therapeutic approach for breast cancer endocrine therapy by promoting hepatic EST activity. Therapeutic strategies targeting the interaction between estrogen and the ER or their downstream signaling have been demonstrated effective in improving the prognosis of HR^+^ breast cancer, and development of new drugs is always a hot topic for endocrine therapy worldwide [48–50]. Although great efforts have been made to develop drugs targeting estradiol synthesis, ER competitive binding, mTOR suppression, and CDK4/6 inhibition, little attention has been paid to modulating estradiol metabolism in the liver [51, 52]. Herein, we provided a novel nano-endocrine drug NCG, which efficiently activated the hepatic EST enzyme and promoted estradiol catabolism. NCG was demonstrated effective in decreasing estradiol levels in mouse liver, serum, and breast tumors, accompanied by reduced tumor sizes comparable to the positive drug TAM. Development of liver-targeting strategies opens a new window for breast cancer endocrine therapy.

Our findings also highlight the significance of EST for breast cancer endocrine therapy. Clinically, EST expression was validated to be positively associated with an improved prognosis of breast cancer [53]. More importantly, EST induction by natural flavonoids, such as quercetin and genistein, was also reported to inhibit the proliferation of breast cancer MCF-7 cells [16, 54]. In contrast, EST inhibition by the persistent environmental pollutants hydroxylated polychlorinated biphenyls increased estradiol bioavailability in target tissues [55]. Similar to previous findings, our study also found that EST expression was reduced in the liver of mice with breast cancer, NCG resulted in a superior effect to monomeric NAR in promoting hepatic EST expression and estradiol inactivation, further suggesting that hepatic EST is an ideal molecular target for the development of endocrine drugs.

Besides, we shed novel light on the biofunction of NAR for breast cancer treatment. NAR is a flavonoid compound found in a number of citrus fruits. Currently, multiple bioactivities of NAR have been reported, including antioxidative, anti-inflammatory, anti-bacterial, anti-coagulation, and anti-cancer effects [56]. For molecular mechanisms, various signaling pathways were reported to be regulated by NAR, such as apoptosis induction, angiogenesis hindrance, and inhibition of Wnt/β-catenin, PI3K/Akt, NF-κB, and TGF-β signaling [57]. With regard to estrogen modulation, NAR has been considered as a weakly estrogenic bioflavonoid that exhibits antiestrogenic activity. NAR was demonstrated to improve ovariectomy-induced osteoporosis, and promoted bone anabolic action through the activation of the osteoblast ER [58]. However, NAR exerted an anti-proliferative and anti-estrogenic effect in ERα-expressing cancer cells, such as hepatoma HepG2 cells, cervix epithelioid carcinoma Hela cells, colon adenocarcinoma DLD-1 cells, and breast cancer MCF-7 cells [59]. Herein, we demonstrated that NAR could decrease estradiol level through triggering hepatic EST expression. More importantly, NAR in NCG nanoparticles showed excellent stability via oral administration and did not cause side effects, such as coagulation disorders, endometrial thickness, or osteoporosis. All these findings suggest that NAR is suitable to be developed as EST agonist or an endocrine prodrug for breast cancer therapy.

In conclusion, our study not only provides a novel nanodrug for breast cancer endocrine therapy by targeting hepatic EST expression, but also highlights the safety of NCG in downregulating estradiol through long term oral administration. The development of NCG as an alternative therapeutic approach is promising, considering its high efficacy and safety, particularly for breast cancer patients with primary or secondary resistance to current endocrine drugs. Our research sheds light on the novel application of nanodrug delivery system in the development of endocrine drugs, and further research is warranted for evaluating the long-term efficacy and safety of NCG in primate breast cancer models and even in clinical trials.

## Materials and methods

### Materials

NAR (purity: 99%) was purchased from Feiyu Biological Co., Ltd (Jiangsu, China). PEG-PCL (PEG, polyethyleneglycol, MW 2000 Da; PCL, Polycaprolactone, MW 6000 Da), CPP-PEG-PCL (CPP, Cell-penetrating peptides RRRRRRRRRRRR), GA-PEG-PCL (GA, galactose, CH_2_OH(CHOH)_4_CHO), Cyanine-5 (Cy5) dye were provided by Ruixi biological Co., Ltd. (Shanxi, China). Phosphate-buffered saline (PBS, pH 7.35), fetal bovine serum (FBS), Dulbecco’s modified eagle medium (DMEM), Roswell Park Memorial Institute 1640 medium (RPMI 1640), trypsin-ethylenediaminetetraacetic acid (Trypsin–EDTA) and penicillin/streptomycin (PS) were bought from Gibco (Carlsbad, CA, USA). Estradiol (E2), 1′-dioctadecyl-3,3,3′,3′-tetramethylindocarbocyanine perchlorate (DiI), Methyl-β-cyclodextrin (MβCD), chlorpromazine (CPZ), hypertonic sucrose, and 5-(*N*-ethyl-*N*-isopropyl)-amiloride (EIPA) were purchased from Sigma-Aldrich Corp. (St. Louis, MO, USA), and estradiol-3-*O*-sulfate (E2-3-*O*-sulfate) was ordered from Meilun Bio-Technology Co., Ltd (Dalian, China). Tamoxifen was ordered from Macklin Co., Ltd (Shanghai, China). 4′,6-Diamidino-2-phenylindole (DAPI), 4% paraformaldehyde (PFA), and Cell counting kit-8 (CCK8) were purchased from Beyotime (Shanghai, China). Lysosome (Lyso)-Tracker™ Deep Red, endoplasmic reticulum (ER)-Tracker™ Red, and mitochondria (Mito)-Tracker™ Red were purchased from Invitrogen (Carlsbad, CA, USA). Methanol and acetonitrile were derived from Merck (Darmstadt, Germany). The deionized water (18 MX) was from a MilliQ water purification system (Millipore, Billerica, MA). HLB solid phase extraction column was sourced from Waters (Milford, MA, USA).

### Preparation of NC, NG, NCG, and Cy5-NCG nanoparticles

To prepare the NCG nanoparticles, we first dissolved 18 mg of PEG-PCL, 1 mg of GA-PEG-PCL, and 1 mg of CPP-PEG-PCL in 1 mL of acetone containing 15 mg of NAR to generate the organic phase. Next, 10 mg of PVP K29/32 was dissolved in 50 mL of dd H_2_O to prepare the aqueous phase, which was then stirred using a magnetic stirrer at 1000 rpm. Subsequently, 0.2 mL of the organic phase was injected into the aqueous phase while maintaining the stirring. The resulting mixture was further stirred for 2–3 min and then subjected to centrifugation to eliminate any unformed precipitate. The resulting nanoparticles were washed three times with deionized water, followed by centrifugation after each wash to remove residual impurities. The preparation procedures for the NC, NG and Cy5-NCG nanoparticles were analogous to that for NCG nanoparticles, except for the use of different PEG-PCL derivatives in the organic phase. For the NC nanoparticles, we dissolved 19 mg of PEG-PCL and 1 mg of CPP-PEG-PCL in 1 mL of acetone containing 15 mg of NAR. For the NG nanoparticles, we dissolved 19 mg of PEG-PCL and 1 mg of GA-PEG-PCL in 1 mL of acetone containing 15 mg of NAR. In case of Cy5-NCG nanoparticles, we dissolved 18 mg of PEG-PCL, 1 mg of GA-PEG-PCL, 1 mg of CPP-PEG-PCL, and 5 mg of Cy5 in 1 mL of acetone to create the organic phase. All other conditions were held constant.

### Nanoparticle characterizations

The morphology and size of NCG were evaluated using a transmission electron microscope (TEM, H-7650, Hitachi, Tokyo, Japan). High-resolution TEM images were captured using a FEI F200S microscope (Waltham, USA) at 200 kV. The zeta potential and particle size distribution of NCG nanoparticles were measured by dynamic light scattering (DLS, Nano-ZS Malvern Instruments, Worcestershire, UK) in dd-H_2_O. The absorption spectra of NCG were obtained using a UV–vis–NIR spectrophotometer (TU-1810, PERSEE, Beijing, China). Additionally, a Fourier transform infrared (FTIR) spectrophotometer (Nicolet iS50, Thermo Fisher Scientific Ltd.) was utilized to record the chemical functions fabricated, which absorbed in the spectral range of 2400 to 4000 cm^−1^. The synthesized NCG was characterized using proton nuclear magnetic resonance (^1^H-NMR) spectroscopy on a Bruker Avance NEO 600 instrument. The physical state of NCG was characterized using differential scanning calorimetry (DSC) analysis on a TA DSC2500 instrument. Each sample (5 mg of free NAR and NCG, respectively) was sealed in a standard aluminum pan. The sample was purged with dry nitrogen gas at a flow rate of 20 mL/min during the DSC analysis. The heating rate was set at 10 °C/min, and the heat flow was recorded within a temperature range of 30 to 400 °C. To assess the stability of NC, NG, and NCG nanoparticles under varying pH conditions, the nanoparticles were exposed to simulated gastric juice with a pH of 1.5 for 2 h, simulated intestinal fluid with a pH of 6.8 for 2 h, and pH 7.35 phosphate-buffered saline (PBS) for 30 days to approximate blood pH. Sedimentation at the bottom of the bottle was observed and documented using a camera, while the particle size of NC, NG, and NCG was measured simultaneously using dynamic light scattering.

The gastric juice was prepared by diluting concentrated hydrochloric acid with water to a concentration of 1 mol/L and adjusting the pH to 1.5. Then, 1 g of pepsin was added to 100 mL of the solution. The intestinal juice was prepared by dissolving 6.8 g of KH_2_PO_4_ in 500 mL of water and adjusting the pH to 6.8 with 0.4% (w/w) NaOH. Then, 1 g of pancreatin was added to 100 mL of the solution.

To evaluate the release kinetics of NAR from NCG, we employed a dialysis-based in vitro method. NCG was first enclosed within a preconditioned dialysis bag (Mw = 8–14 kDa) and immersed in a beaker containing simulated gastric acid for 2 h, followed by simulated intestinal fluid for another 2 h. The bag was then transferred to a beaker filled with 40 mL of pH 7.4 phosphate-buffered saline (PBS) containing 0.5% (w/v) Tween 80, and maintained for 44 h on a rotary shaker at 37 °C and 70 rpm. At predetermined intervals, aliquots of the release medium were withdrawn and replaced with an equal volume of fresh medium to ensure sink conditions. The dissolved samples were then collected in 2 mL beakers for further analysis. The concentration of NAR was determined by measuring the absorbance at 260 nm and correlating it with an established UV–Vis standard curve.

UV–Vis–NIR spectroscopy was used to determine the drug loading (DL) and encapsulation efficiency (EE) of naringenin (NAR) (λ max = 360 nm). The DL and EE values were then calculated using the following equations: DL (%) = Wa/Wb × 100; EE (%) = Wa/(Wa + Wc) × 100. Here, Wa, Wb, and Wc correspond to the mass of encapsulated NAR, the total weight of NCG, and the mass of free NAR, respectively.

### Cell culture

WRL-68, MCF-7, Caco-2, HUVEC, E0771, and HBL-100 were obtained from Nanjing Keygen Biotech. WRL-68, MCF-7, Caco-2, and HUVEC cells were cultured in DMEM cell culture medium supplemented with 10% fetal bovine serum and 1% penicillin/streptomycin, and E0771 and HBL-100 cells were cultured in the same formulation of RPMI-1640 cell culture media. All cells were maintained at 37 °C in a humidified atmosphere containing 5% CO_2_ with regular sub-culturing.

### In vitro cytotoxicity analysis

WRL-68, MCF-7, E0771, Caco-2, HUVEC, and HBL-100 cells were seeded into 96-well plates at a density of approximately 5 × 10^3^ cells per well. NAR, NC, NG, and NCG (at equal concentrations of NAR) were added to the wells, and a control group was also included. After drug addition, the plates were placed in a CO_2_ incubator and incubated at 37 °C with 5% CO_2_ for 24 h. CCK-8 reagent was diluted according to the manufacturer’s instructions (Beyotime Biotechnology, C0038), and the old medium was aspirated from the wells and replaced with fresh medium containing the diluted CCK-8 reagent. After a further 2 h incubation at 37 °C, the absorbance was measured at a wavelength of 450 nm using a microplate reader (Biotek, EON).

### Cellular uptake detection

WRL-68, MCF-7, E0771, Caco-2, HUVEC, and HBL-100 cells were seeded separately into 12-well plates containing complete culture medium at a density of approximately 1 × 10^5^ cells per well. Subsequently, the plates were incubated for the specified nanoparticles at 37 °C and 5% CO_2_ in a CO_2_ incubator. Upon completion of the incubation period, cells were rinsed thrice with PBS, fixed with 4% paraformaldehyde, and subjected to DAPI staining for visualization of the cellular nuclei. The acquired cellular images will undergo further analysis employing a confocal laser scanning microscope (Zeiss LMS-780, Thornwood, New York). For the evaluation of liver targeting efficacy, an excess of free GA was introduced half an hour prior to the experiment, thereby inducing ASGPR saturation.

### Intestinal barrier permeability assay

To evaluate the ability of NCG to cross the intestinal barrier, we established an in vitro intestinal barrier model based on previous literature [60]. The model was constructed as follows: Caco-2 cells were seeded onto the PET membrane of the Transwell upper chamber at a density of 1 × 10^5^ cells/cm^2^. When the transepithelial electrical resistance (TEER) reached over 150 Ω/cm^2^, WRL-68 cells were seeded in the lower chamber at 1 × 10^5^ cells/cm^2^ density and incubated at 37 °C in a 5% CO_2_ atmosphere for 24 h. Subsequently, NAR and NCG drugs (with an equivalent concentration of 100 nmol/L for NAR) were added to the upper chamber, and the Transwell was returned to the incubator for 10, 30, 60, and 120 min. At specified time points, the Caco-2 cells in the upper chamber and the WRL-68 cells in the lower chamber were fixed and stained with DAPI for nuclear visualization. The distribution of NAR and NCG on both sides of the PET membrane was observed using the Z-axis scanning function of a confocal laser scanning microscope (Zeiss LMS-780, Thornwood, New York), enabling further analysis of drug penetration within the intestinal barrier model.

### FRET assay

Förster Resonance Energy Transfer (FRET) assay was applied to evaluate the integrity of NCG in Caco-2 and WRL-68 cells. We encapsulated C6 and DiI fluorophores within NCG and exposed them to Caco-2 and WRL-68 cells, respectively. The cells were incubated at 37 °C in a 5% CO_2_ atmosphere for 1 h and 2 h. Following incubation, the cells were washed thrice with cold PBS, fixed with 4% PFA, stained with DAPI, and analyzed using a confocal laser scanning microscope for evaluation.

### Cellular endocytosis and distribution assay

Caco-2 and WRL-68 cells were cultured on cover slips in a 12-well plate for 24 h. In order to investigate the endocytic pathways of NCG, cells were treated with endocytosis-mediated clathrin-dependent inhibitor CPZ, caveolin-mediated endocytosis inhibitor MβCD, and macropinocytosis inhibitor EIPA for a duration of 30 min. Subsequently, NCG was introduced into DMEM culture medium containing 10% FBS and co-incubated with cells for 30 min. To explore the subcellular distribution of NCG in Caco-2 and WRL-68 cells, following the 30 min co-incubation of NCG with cells, mitochondria tracker, lysosome tracker, and endoplasmic reticulum tracker were individually introduced and co-incubated with cells according to the manufacturer’s specifications. Following incubation, cells were washed with ice-cold PBS and fixed with 4% paraformaldehyde. Cell nuclei were stained with DAPI, and laser confocal microscopy was utilized to assess the uptake of NCG in various treatment groups, as well as its co-localization with distinct cellular sub-organelles.

### Luciferase reporter assays in vitro

To investigate the effects of different drugs on the EST expression, the luciferase reporter gene assay was conducted. The EST promoter fragment was amplified and subsequently cloned into the pGL3 basic vector (LvPG04, GeneCopoeia, China). WRL-68 cells were transfected with the EST luciferase reporter plasmid (5 µg/well) in 6-well plates. Following 48 h post-transfection, the cells were seeded in a 96-well plate, and the drugs NAR, NC, NG, and NCG were administered at respective NAR equivalent concentrations (0, 1, 5, 10, 50, 100, 200 nmol/L). After a 24 h drug treatment, the supernatant was collected, and luciferase activity was assessed. Promoter activity was determined using the Secrete-Pair Dual Luminescence Detection Kit (LF001, GeneCopoeia, China), following the manufacturer's instructions. Results were normalized to the activity of renilla luciferase. All transfection experiments were performed in triplicate and independently repeated three times.

### Zebrafish experiments

In order to observe the distribution of different NAR formulations in zebrafish, we exposed zebrafish larvae (7 days post-fertilization; dpf) to NAR or NCG at concentrations of 50, 100, or 200 nmol/L. Following exposure durations of 10, 30, or 60 min, the zebrafish were anesthetized and subsequently collected for microscopic imaging utilizing a Leica DMi8 microscope (Leica Microsystems, Wetzlar, Germany). To assess the inhibitory potential of NCG on breast cancer cell proliferation, a zebrafish breast cancer xenotransplantation model was developed. E0771 cells were specifically labeled with 5 μmol/L DiI and microinjected into zebrafish embryos at 48 h post-fertilization. The zebrafish harboring E0771 cells were subsequently distributed into 96-well plates (10 fish per well) and cultured in a medium supplemented with 40 μmol/L estradiol, excluding the control group. Interventions with NAR, NC, NG, and NCG were then administered at a 50 nmol/L concentration for each compound. Following a 48 h incubation period, the growth and metastasis of E0771 cells were observed using an inverted fluorescence microscope (Nikon Eclipse C1, Tokyo, Japan).

### Mice experiments

C57BL/6 female mice (6–8 weeks old) were procured from the Guangdong Provincial Experimental Animal Center (Guangzhou, China) and maintained under a 12-h light–dark cycle, a consistent room temperature of 20–22 °C, and a relative humidity of 30–70% (SCXK-2020-100). The C57BL/6 female mice were allowed to acclimate to these conditions for a minimum of 7 days before experimentation commenced. Following the stress treatment period, an in situ breast cancer model was established by injecting 1 × 10^6^ ER-positive E0771 breast cancer cells suspended in a 100 μL PBS-Matrigel (1:1) mixture into the fourth mammary fat pad of female C57BL/6 mice. The mice were then randomly assigned to eight groups (n = 6 per group), comprising a control group, Saline group, NAR L (10 mg/kg), NAR H (20 mg/kg), TAM, NG (10 mg/kg), NC (10 mg/kg), and NCG (10 mg/kg) groups. The corresponding treatments were administered orally everyday. Tumor dimensions were measured using a vernier caliper every 3 days, and tumor volume was estimated employing the formula (length × width^2^/2). After a 14-day treatment period, all mice were sacrificed, and tumor volume and weight were assessed. All animal studies and experimental protocols were approved by the Animal Ethics Committee of the Guangdong Provincial Hospital of Chinese Medicine (2021074).

### In vivo NIR imaging

To assess the intestinal barrier permeability and liver-targeting properties of NCG, we prepared nanocarriers and labeled them with Cy5. Mice were orally administered with Cy5, Cy5-NC, Cy5-NG, and Cy5-NCG (Cy5 = 1 mg/kg) and subsequently subjected to whole-body imaging using an in vivo imaging system at various time points (n = 3). The distribution of the fluorescence signal was observed under excitation at 650 nm and emission at 700 nm wavelength. Additionally, a subset of mice was sacrificed to examine the fluorescence signal in major organs, including the stomach, duodenum, jejunum, ileum, liver, heart, spleen, lung, kidney, and tumor. The fluorescence signal was then quantified using the IndiGo software.

### In vivo pharmacokinetics analysis

To perform pharmacokinetic studies using Sprague–Dawley (SD) rats, the animals were randomly assigned to four groups including NAR, NC, NG, and NCG. Each rat received the corresponding treatment orally at an equivalent dose of 6.9 mg/kg NAR. Blood samples (300 μL) were collected from the orbital socket of the rats (n = 6/group) at 0.083, 0.5, 1, 2, 4, 6, 12, 24, and 48 h post-administration. The NAR levels in these samples were quantified using liquid chromatography-tandem mass spectrometry (LC–MS/MS). Furthermore, the Drug and Statistics (DAS) 2.0 platform was employed to determine pharmacokinetic parameters, including elimination half-life (T1/2), peak brain/blood quotients of concentration and time (QCT) levels (Cmax), time to reach peak level (Tmax), and area under the curve.

### Western blot

Fresh frozen liver samples were sectioned into small pieces and homogenized in a standard lysis buffer containing 1 mmol/L protease inhibitor. The homogenized suspension was subsequently centrifuged to isolate the supernatant, which was preserved at − 80 °C for future use. The protein concentration in the supernatant was ascertained using the bicinchoninic acid (BCA) protein assay and adjusted accordingly. For protein electrophoresis, the adjusted supernatant was combined with 1×  SDS-PAGE buffer and subjected to electrophoretic separation. The resolved proteins were then transferred to a polyvinylidene fluoride (PVDF) membrane, which was blocked with a blocking solution (5% non-fat milk) to prevent nonspecific binding. The membrane was incubated with primary antibodies EST (ProteinTech, 12522-1-AP), overnight at 4 °C, followed by washing and incubation with the corresponding secondary antibodies. Protein bands were visualized using the ChemiDoc XRS^+^ Imaging System (Bio-Rad) and quantified employing ImageJ or other image processing software.

### Immunofluorescence assay

Immunofluorescence was employed to examine the expression of the EST in WRL-68 cells. WRL-68 cells were seeded on a 12-well plate slide. Following drug treatment, the cells were washed thrice with PBS for 5 min each. The cells were then fixed with 4% paraformaldehyde and blocked with 10% bovine serum. To examine the expression of the EST in the liver, frozen liver sections (4 µm thick) were prepared and submerged in ultrapure water for 2 min. The slides were subsequently fixed in cold acetone at 20 °C for 10 min and then incubated with a blocking buffer for 30 min. After washing the slides three times with PBS at room temperature for 5 min each, they were incubated with primary antibody against EST (ProteinTech, 12522-1-AP) overnight at 4 °C. The slides were rewashed three times with PBS for 5 min each, followed by incubation with Alexa Fluor 488 or Alexa Fluor 555-conjugated anti-mouse IgG at a 1:200 dilution. After stained with 4′,6-diamidino-2-phenylindole (DAPI, Sigma) for nuclear visualization, the slides were imaged using a confocal laser scanning microscope (Zeiss LMS-780, Thornwood, New York).

### Detection of E2 and E2-3-*O*-sulfate level

To investigate the effects of NAR and NCG on estrogen metabolism, WRL-68 cells were seeded in 12-well plates and treated with NAR and NCG for 24 h, respectively. Subsequently, the cells were incubated with Hanks’ Balanced Salt Solution containing 40 μmol/L E2 at 37 °C for 4 h. The extracellular medium was collected and analyzed using UPLC-QTOF/MS. As for blood serum, tumors, and liver samples, tissues were homogenized in 600 μL of ice-cold extraction solvent (90% methanol/water) and incubated on ice for 30 min. After centrifugation at 12,000×*g* for 15 min at 4 °C, the supernatant was gathered, transferred to a new Eppendorf tube, and dried in a vacuum concentrator. Subsequently, HLB solid-phase extraction (Waters, Milford, MA) was employed to desalt the blood serum. Equal aliquots of 100 μL serum were loaded onto the solid-phase extraction cartridge and eluted with 1 mL of acetonitrile. The acetonitrile eluate was vacuum-dried and reconstituted with 80 μL of acetonitrile/water (1:1). An ACQUITY UPLC-QTOF/MS System (Waters, Milford, MA) equipped with an ACQUITY BEH C18 Column (2.1 × 50 mm, 2.6 µm; Waters, Milford, MA) was employed for the analysis. The mobile phases consisted of 0.1% formic acid (mobile phase A) and 0.1% formic acid in acetonitrile (mobile phase B). The flow rate was set at 0.25 mL/min. The gradient elution program followed a sequence of 25% B from 0 to 1 min, an increase from 25 to 85% B between 1 and 2.8 min, and a decrease from 85 to 25% B between 2.8 and 4 min.

### In vivo biocompatibility assays

Upon completion of the treatment period, blood samples were collected from each group of mice for the analysis of blood cells, biochemical markers, and coagulation factors. The heart, liver, spleen, lung, kidney, and uterus were fixed in 4% paraformaldehyde (PFA) and sectioned into 5 µm-thick slices. For pathological examination, tissue sections were stained with hematoxylin and eosin (H&E) and assessed for potential organ damage. The endometrial thickness was measured to evaluate the impact of estrogen level fluctuations on the uterus.

### Micro computed tomography

Specimens were scanned using Bruker Micro-CT Skyscan 1276 system (Kontich, Belgium). Scan settings are as follows: voxel size 6.534165 μm, medium resolution, 70 kV, 200 μA, 0.25 mm Al filter and integration time 350 ms. Density measurements were calibrated to the manufacturer’s calcium hydroxyapatite (CaHA) phantom. Analysis was performed using the manufacturer’s evaluation software. Reconstruction was accomplished by NRecon (version 1.7.4.2). 3D images were obtained from contoured 2D images by methods based on distance transformation of the grayscale original images (CTvox; version 3.3.0). 3D and 2D analysis were performed using software CT Analyser (version 1.20.3.0).

### Statistical analysis

Data were presented as mean ± standard deviation (SD). Student’s *t*-test analysis or one-way ANOVA was applied. Post hoc testing of dose-dependent data was conducted using Dunnett’s test, while Bonferroni's post hoc test was used for other data. Repeated measures analysis of variance was used for repeated measures data. Nonparametric tests were utilized for data that were not normally distributed. A p-value of < 0.05 was considered statistically significant. Statistical analysis was performed using SPSS software (IBM SPSS Statistics, version 29.0).

### Supplementary Information

**Additional file 1: Figure S1.** ^1^H NMR of (A) GA-PEG-PCL and (B) CPP-PEG-PCL. (C) Particles size distributions and particle size of Cy5-NCG. (D) Visual graph of NG and NCG stability at pH 7.35 at 1st day and 30th days. **Figure S2.** (A) The ratio of NAR concentration at the end of the incubation to the initial concentration in the upper chamber of the Transwell. (B) Statistical values of fluorescence intensity of NAR and NCG in WRL-68 cells in the lower chamber of Transwell (n = 6). Data are presented as mean ± SD, ns:* p* > *0.05*; ***: *p* < *0.001* indicates a comparison between the two groups. **Figure S3.** Evaluation of cytotoxicity in RAW 264.7 (A), HBL-100 (B), HUVEC (C), and MCF-7 (D) cells for various NAR formulations (n = 3). Assessment of the ability of multiple NAR formulations to activate EST genes at 4 h (E) and 12 h (F) using the dual luciferase reporter gene assay (n = 3). Data are presented as mean ± SD, ns:* p* > *0.05*; *: *p* < *0.05*; **: *p* < *0.01*; ***: *p* < *0.001* indicates a comparison between the two groups. **Figure S4.** Statistical analysis of fluorescence intensity of EST enzyme expression levels in mouse liver tissue, assessed by immunofluorescence across groups (n = 6). Data are presented as mean ± SD, *ns: p* > *0.05*, *p < *0.05*, ***p < *0.001* indicates a comparison between the two groups; ^##^*p* < *0.01*, ^###^*p* < *0.001* compared to the Control group. **Table S1.** NAR pharmacokinetic parameters following oral administration of various NAR formulations.

## Data Availability

The datasets used and analysed during the current study are available from the corresponding author on reasonable request.
