# Supplementary material for: Developing liver-targeted naringenin nanoparticles for breast cancer endocrine therapy by promoting estrogen metabolism
Source: J Nanobiotechnology. 2024 Mar 19;22:122. doi: 10.1186/s12951-024-02356-0 (PMC10953142; doi:10.1186/s12951-024-02356-0)
Supplement: Supplementary file 1 — Additional file 1: Figure S1. 1H NMR of (A) GA-PEG-PCL and (B) CPP-PEG-PCL. (C) Particles size distributions and particle size of Cy5-NCG. (D) Visual graph of NG and NCG stability at pH 7.35 at 1st day and 30th days. Figure S2. (A) The ratio of NAR concentration at the end of the incubation to the initial concentration in the upper chamber of the Transwell. (B) Statistical values of fluorescence intensity of NAR and NCG in WRL-68 cells in the lower chamber of Transwell (n = 6). Data are presented as mean ± SD, ns: p > 0.05; ***: p < 0.001 indicates a comparison between the two groups. Figure S3. Evaluation of cytotoxicity in RAW 264.7 (A), HBL-100 (B), HUVEC (C), and MCF-7 (D) cells for various NAR formulations (n = 3). Assessment of the ability of multiple NAR formulations to activate EST genes at 4 h (E) and 12 h (F) using the dual luciferase reporter gene assay (n = 3). Data are presented as mean ± SD, ns: p > 0.05; *: p < 0.05; **: p < 0.01; ***: p < 0.001 indicates a comparison between the two groups. Figure S4. Statistical analysis of fluorescence intensity of EST enzyme expression levels in mouse liver tissue, assessed by immunofluorescence across groups (n = 6). Data are presented as mean ± SD, ns: p > 0.05, *p < 0.05, ***p < 0.001 indicates a comparison between the two groups; ##p < 0.01, ###p < 0.001 compared to the Control group. Table S1. NAR pharmacokinetic parameters following oral administration of various NAR formulations. [file 12951_2024_2356_MOESM1_ESM.docx]

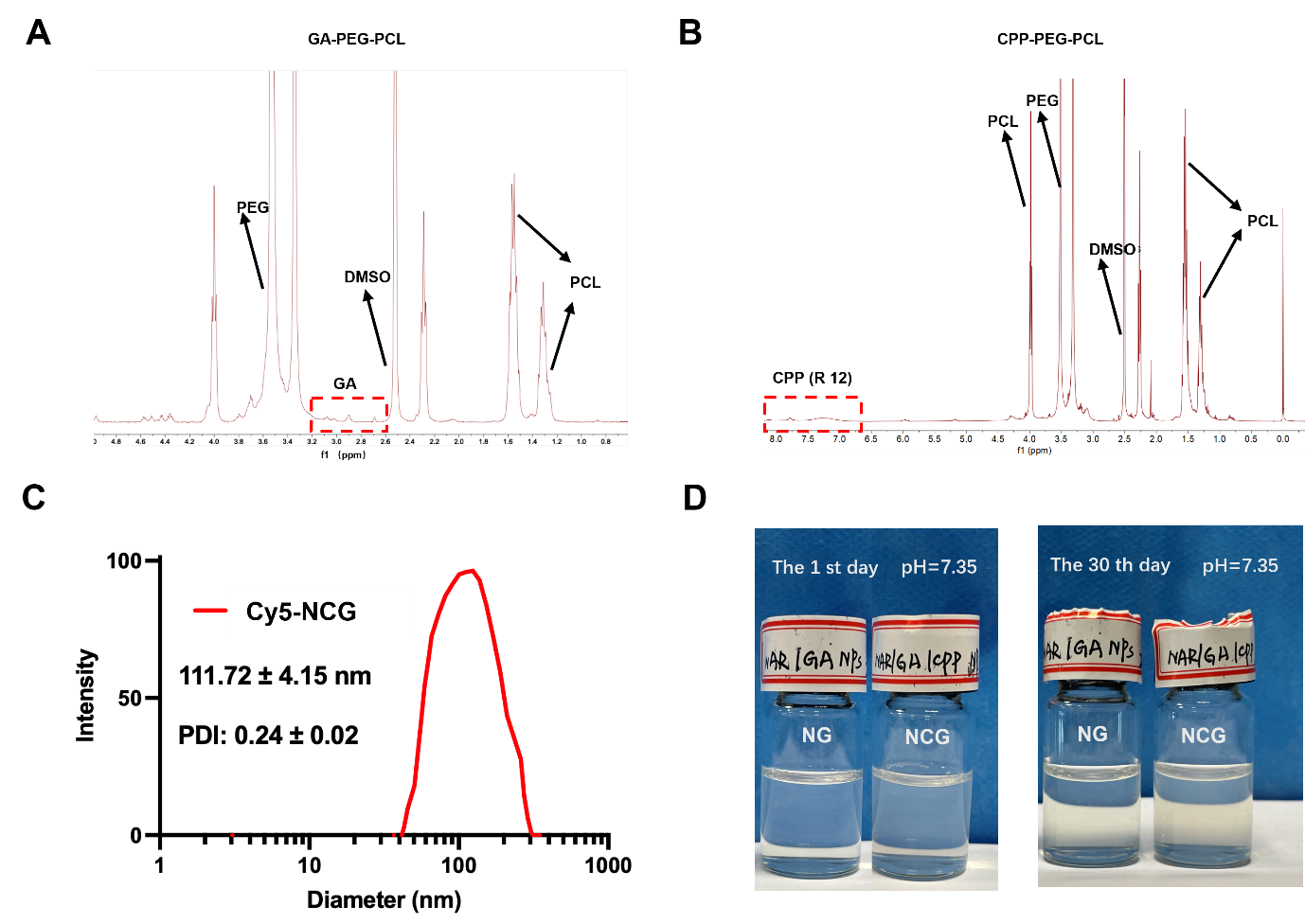


**Figure S1.** ^1^H NMR of (A) GA-PEG-PCL and (B) CPP-PEG-PCL. (C) Particles size distributions and particle size of Cy5-NCG. (D) Visual graph of NG and NCG stability at pH 7.35 at 1^st^ day and 30^th^ days.


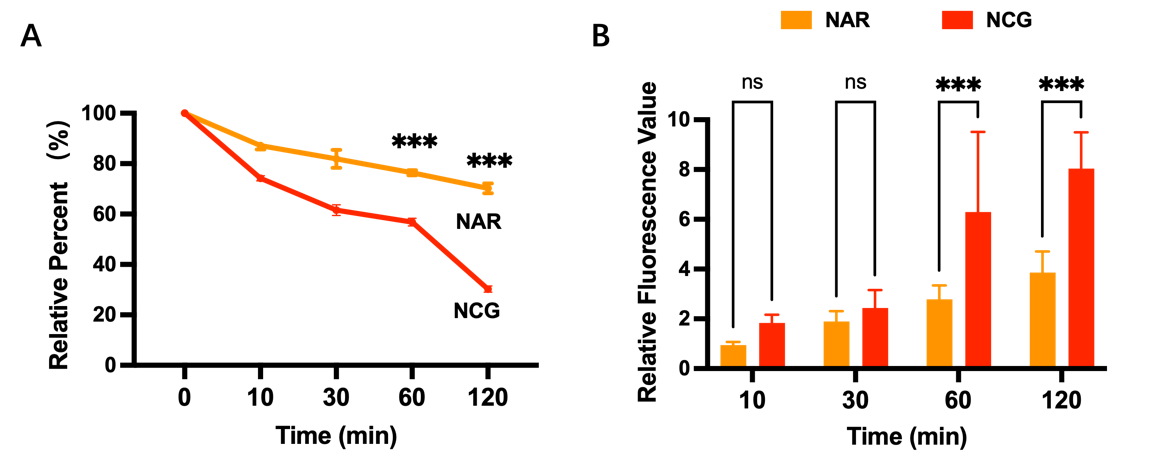


**Figure S2.** (A) The ratio of NAR concentration at the end of the incubation to the initial concentration in the upper chamber of the Transwell. (B) Statistical values of fluorescence intensity of NAR and NCG in WRL-68 cells in the lower chamber of Transwell (n=6). Data are presented as mean ± SD, *ns: p>0.05;* ****: p<0.001* indicates a comparison between the two groups*.*


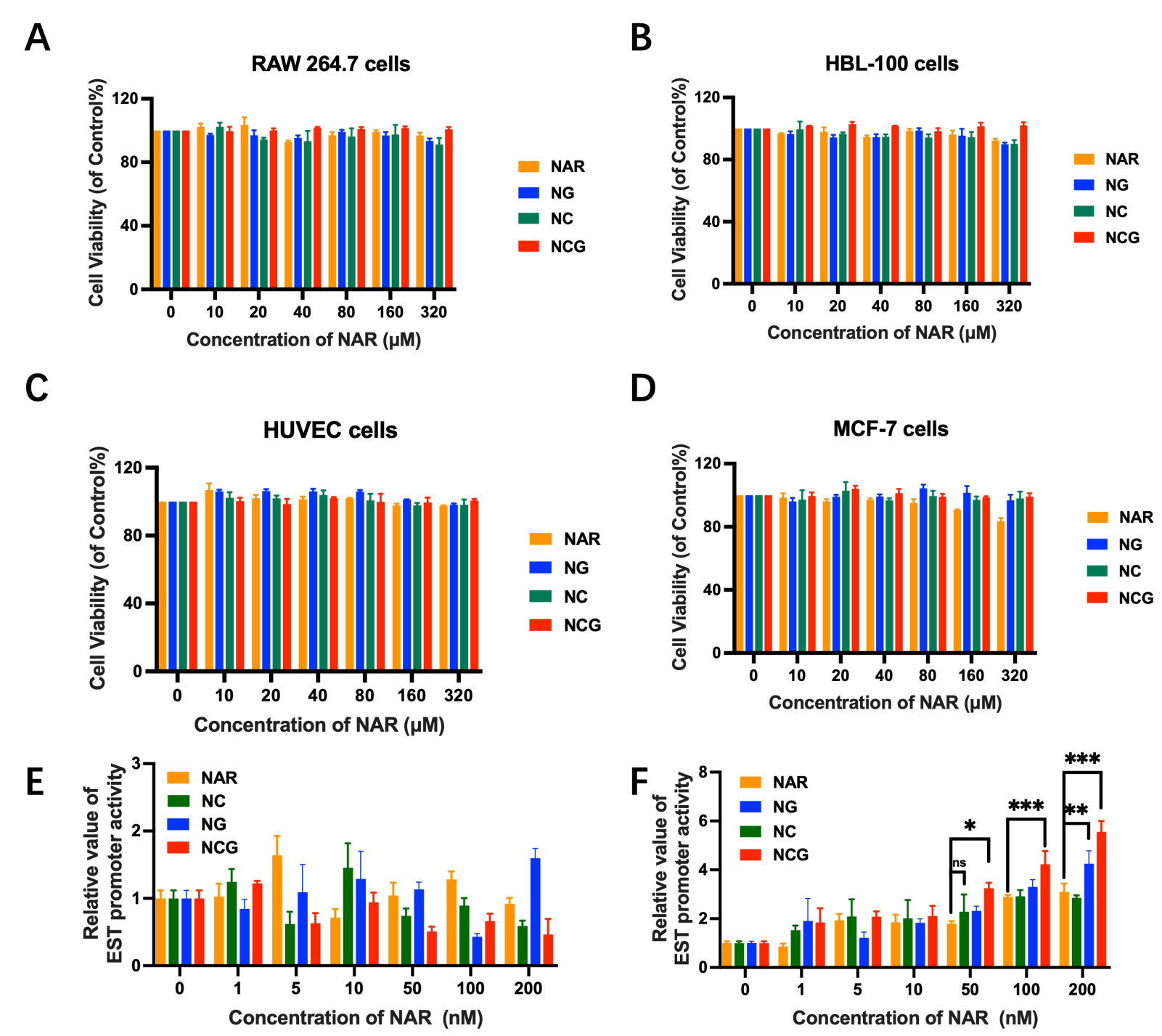


**Figure S3.** Evaluation of cytotoxicity in RAW 264.7 (A), HBL-100 (B), HUVEC (C), and MCF-7 (D) cells for various NAR formulations (n=3). Assessment of the ability of multiple NAR formulations to activate EST genes at 4 h (E) and 12 h (F) using the dual luciferase reporter gene assay (n=3). Data are presented as mean ± SD, *ns: p>0.05; *: p<0.05; **: p<0.01**; ***: p<0.001* indicates a comparison between the two groups*.*


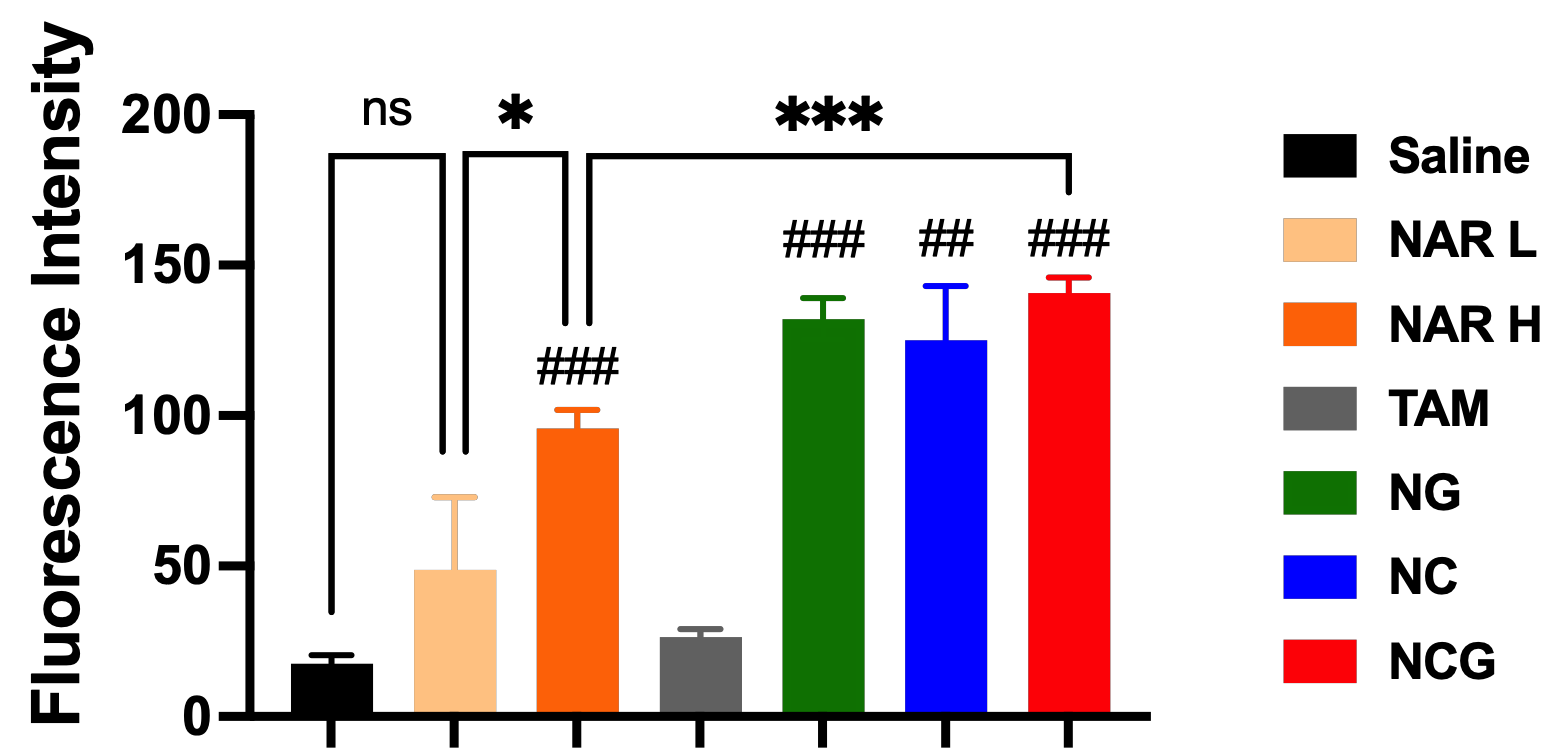


**Figure S4.** Statistical analysis of fluorescence intensity of EST enzyme expression levels in mouse liver tissue, assessed by immunofluorescence across groups (n=6). Data are presented as mean ± SD, *ns: p>0.05, *p<0.05, ***p<0.001* indicates a comparison between the two groups; *^##^p<0.01, ^###^p<0.001* compared to the Control group.

**Table S1.** NAR pharmacokinetic parameters following oral administration of various NAR formulations.

| Parameters (n=6) | NAR | NC | NG | NCG |
| --- | --- | --- | --- | --- |
| C_max_ (ug/L) | 87.99 ± 14.81 | 290.80 ± 71.80 | 315.49 ± 1.53 | 507.04 ± 13.28 |
| t_max_ (h) | 2.00 ± 0.00 | 1.00 ± 0.00 | 2.00 ± 0.00 | 1.00 ± 0.00 |
| t_1/2_ (h) | 2.74 ± 0.34 | 4.51 ± 1.20 | 4.99 ± 1.21 | 5.06 ± 1.52 |
| AUC_0-24_ (ug/L*h) | 360.98 ± 144.81 | 657.15 ± 92.29 | 1436.59 ± 69.71 | 3393.19 ± 245.41 |
| F | 100.00 % | 182.54 % | 397.96 % | 939.99 % |
